# Glass‐Crystallized Luminescence Translucent Ceramics toward High‐Performance Broadband NIR LEDs

**DOI:** 10.1002/advs.202105713

**Published:** 2022-01-24

**Authors:** Guojun Zheng, Wenge Xiao, Jianhong Wu, Xiaofeng Liu, Hirokazu Masai, Jianrong Qiu

**Affiliations:** ^1^ State Key Lab of Modern Optical Instrumentation College of Optical Science and Engineering Zhejiang University Hangzhou 310027 P. R. China; ^2^ School of Materials Science and Engineering Zhejiang University Hangzhou 310027 P. R. China; ^3^ National Institute of Advanced Industrial Science and Technology Osaka 563‐8577 Japan; ^4^ CAS Center for Excellence in Ultra‐intense Laser Science Shanghai Institute of Optics and Fine Mechanics Chinese Academy of Sciences Shanghai 201800 P. R. China

**Keywords:** broadband light sources, Cr^3+^, garnets, glass crystallization, light conversion

## Abstract

Near‐infrared (NIR) phosphor‐converted light‐emitting diodes (pc‐LEDs) are newly emergent broadband light sources for miniaturizing optical systems like spectrometers. However, traditional converters with NIR phosphors encapsulated by organic resins suffer from low external quantum efficiency (EQE), strong thermal quenching as well as low thermal conductivity, thus limiting the device efficiency and output power. Through pressureless crystallization from the designed aluminosilicate glasses, here broadband Near‐infrared (NIR) emitting translucent ceramics are developed with high EQE (59.5%) and excellent thermal stability (<10% intensity loss and negligible variation of emission profile at 150 °C) to serve as all‐inorganic visible‐to‐NIR converters. A high‐performance NIR phosphor‐converted light emitting diodes is further demonstrated with a record NIR photoelectric efficiency (output power) of 21.2% (62.6 mW) at 100 mA and a luminescence saturation threshold up to 184 W cm^−2^. The results can substantially expand the applications of pc‐LEDs, and may open up new opportunity to design efficient broadband emitting materials.

## Graphical abstract

A cost‐effective and pressureless method based on glass crystallization is proposed to develop broadband near‐infrared (NIR) emitting translucent ceramics with high quantum efficiency and excellent thermal stability, which enables the fabrication of all‐inorganic NIR phosphor‐converted light emitting diodes with high conversion efficiency and high output power. These results provide new possibilities for the development of efficient luminescence materials for non‐visible optical applications.

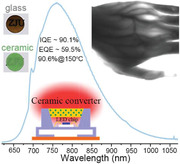

## 1 Introduction

Broadband light sources are essential to a variety of optical and photonic systems such as near‐infrared (NIR) spectrometers,^[^ ^1^ ^]^ optical coherence tomography systems,^[^ ^2^ ^]^ and solar simulators.^[^ ^3^ ^]^ In particular, with the unprecedented penetration of portable and hand‐held devices like smartphones, there is growing interest in miniaturizing the desktop NIR spectrometer to be a non‐invasive and point‐of‐care diagnostic technology for food analysis, health monitoring, and material identification.^[^ ^4^ ^]^ To enable such a transformation, one of the major obstacles is the lack of compact broadband near‐infrared (NIR) light sources with high efficiency, high power, and affordable price.^[^ ^4^, ^5^ ^]^ Instead of NIR (superluminescent) light‐emitting diodes (LEDs) with a small full‐width at half maximum (FWHM < 100 nm),^[^ ^6^ ^]^ phosphor‐converted LEDs (pc‐LEDs) based on blue InGaN LED chips have recently been demonstrated to be an excellent candidate, since extra‐broadband emission with FWHM > 300 nm can be readily obtained by embedding several kinds of NIR phosphors into cured organic resins as light converters.^[^ ^7^ ^]^ As compared to red/green/blue (RGB) visible emission, NIR emission, especially the broadband one, intrinsically suffer from low internal quantum efficiency (IQE) and strong thermal quenching due to the small energy gap between the two related energy states and the strong interaction of the excited state with its surroundings,^[^ ^6^, ^8^ ^]^ thereby posing a greater challenge to efficient NIR‐emitting materials.

Trivalent chromium ion (Cr^3+^)^[^ ^9^ ^]^ has shown its superiority to other activator ions^[^ ^10^ ^]^ in emitting far‐red/NIR light of 650–1100 nm upon blue light excitation. For example, high IQE (>90%) was achieved through cation substitution^[^ ^9c,d^ ^]^ and optimizing synthesis strategy;^[^ ^11^ ^]^ single‐phase extra‐broadband emission with FWHM > 200 nm was also realized by exploring host crystals allowing multiple‐site occupation of Cr^3+^.^[^ ^12^ ^]^ Nevertheless, the fundamental problem for Cr^3+^ activated phosphors is the small absorption cross‐section (10^−19^–10^−20^ cm^2^) of Cr^3+^ as a result of the parity‐forbidden nature of intraconfigurational *d–d* transitions (^4^A_2_ → ^4^T_1_/^4^T_2_).^[^ ^13^ ^]^ Increasing the doping concentration,^[^ ^11^, ^14^ ^]^ and introducing the odd‐parity crystal field by lattice distortion,^[^ ^15^ ^]^ can expectedly improve the light absorption efficiency (AE), but inevitably impose serious adverse effects on IQE and thermal stability because of the enhanced (thermally activated) concentration quenching and/or the reduced structural rigidity.^[^ ^16^ ^]^ Another knotty problem for traditional NIR pc‐LEDs with phosphor powders embedded in organic resin is the massive heat accumulation resulting from large quantum defect (>40%) in down‐converting blue light into NIR one and the very low thermal conductivity (≈0.2 W m^−1^ K^−1^) of organic binders, which fundamentally limit the performance of the pc‐LED devices.^[^ ^17^ ^]^

Here, we developed a Cr^3+^ activated broadband NIR translucent ceramic to serve as an all‐inorganic converter for circumventing these problems. Traditional powder sintering for the densification of ceramics relies on high pressure and high vacuum techniques, which is expensive and also limited to the preparation of RGB ceramics for high‐power white light sources.^[^ ^17^, ^18^ ^]^ Instead, we adopted a much simpler and pressureless method, that is, full crystallization of amorphous materials (glasses) into crystalline ones (ceramics),^[^ ^19^ ^]^ which has succeeded in developing transparent ceramics without cubic symmetry via introducing structural disorder^[^ ^20^ ^]^ and efficient RGB multi‐phase translucent composites.^[^ ^21^ ^]^ Considering the large flexibility of garnet crystals in composition,^[^ ^22^ ^]^ we selected the well‐known Y_3_Al_5_O_12_ (YAG) garnet as the starting material. Then we designed a series of garnet solid solutions via dual‐function cation cosubstitution of Ca^2+^—Si^4+^ for Y^3+^—Al^3+^, by which narrowband far‐red emission of Cr^3+^ was largely regulated to broadband NIR one due to the weakened crystal field of the octahedrally coordinated Cr^3+^,^[^ ^23^ ^]^ and more importantly, YAG:Cr^3+^ can be engineered into a glass‐forming material.^[^ ^24^ ^]^ Benefitting from the single garnet phase with high‐quality crystallinity and the reduced light scattering within translucent ceramics,^[^ ^17^, ^18^ ^]^ we achieved a record external QE (EQE) of 59.5% accompanied by high IQE (90.1%) and excellent thermal stability, thus enabling a proof‐of‐concept demonstration of all‐inorganic pc‐LEDs with high‐power and high‐efficiency broadband NIR emission.

## 2 Results

### 2.1 Glass Crystallization and Structural Characterization

Transparent glass precursors with the compositions of Y_3−_ *_x_* Ca*_x_* Al_5−_ *_x_*_−_ *_y_* Cr*_y_* Si*_x_* O_12_ (*x* = 0.0–1.4, *y* = 0.00–0.08) were obtained using an aerodynamic levitation furnace equipped with a laser heating system (see Experimental Section and Figure S1, Supporting Information), which enables containerless melting and rapid cooling at high temperature (up to 2000 °C) without contamination.^[^ ^19^, ^21^ ^]^ To transform YAG into a glass‐forming material, we introduced the glass network former SiO_2_ to establish the required interconnectivity.^[^ ^24^ ^]^ Then, CaO was added to ensure the charge balance within the resulting garnet crystals by cosubstitution of (Ca^2+^—Si^4+^) for (Y^3+^—Al^3+^),^[^ ^23^, ^25^ ^]^ avoiding unexpected defects as well. After heat‐treated for 10 h above the crystallization temperature (Figure S2, Supporting Information) in a reducing atmosphere, the glass precursors were fully crystallized into translucent polycrystalline ceramics with single garnet phase when *x* ≤ 1, beyond which the impurity phases begin to occur (Figure S3, Supporting Information). Therefore, we focused the present study on Y_2_CaAl_4_SiO_12_:Cr^3+^ (YCAS:Cr^3+^).

**Figure 1:**
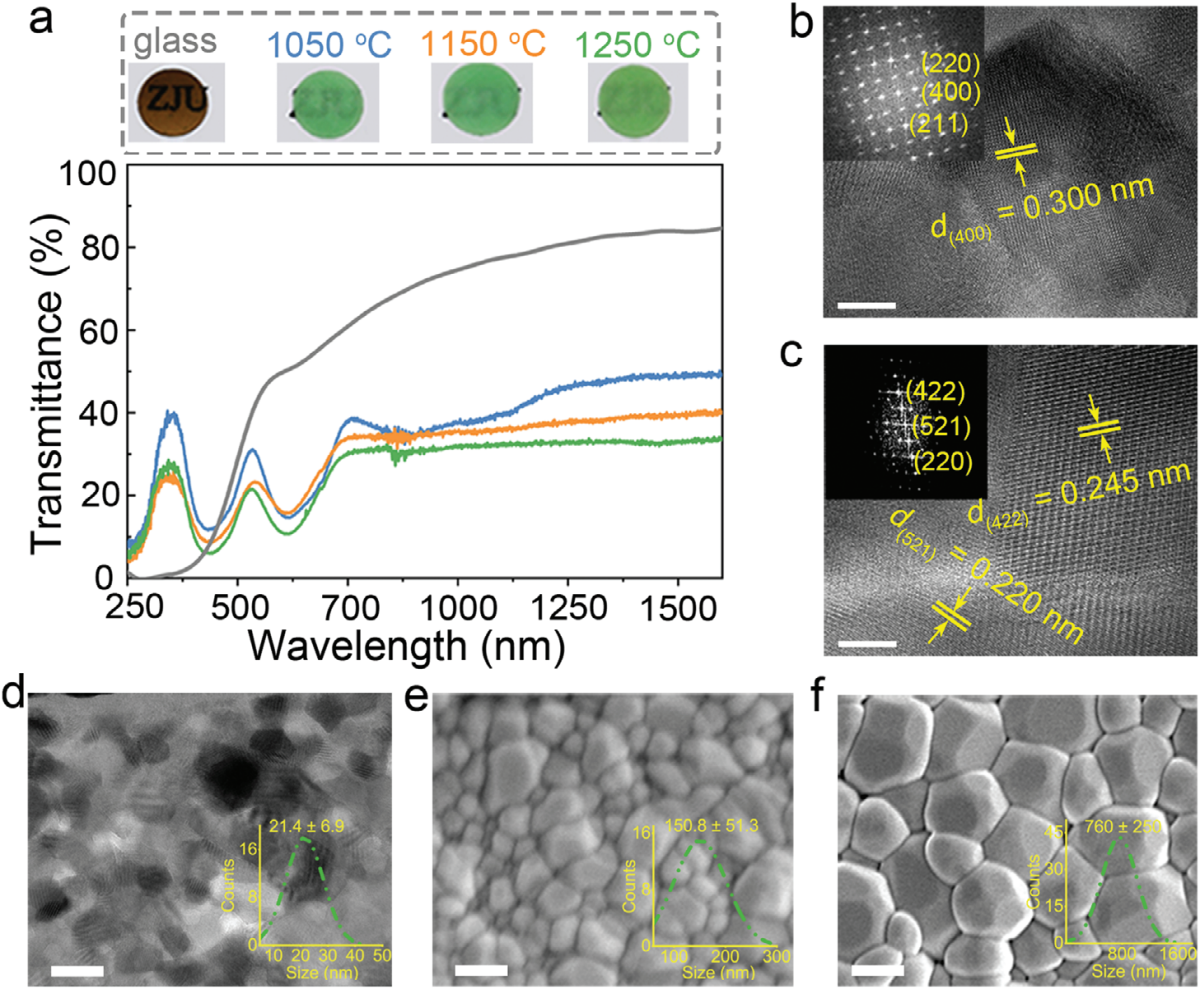
Glass crystallization and structural characterization. (a) The transmission spectra of YCAS:0.04Cr^3+^ glass and ceramics annealed at different temperatures. The insets on the top are the photographs of the as‐prepared samples (0.4 mm in thickness and 4–5 mm in diameter). The absorption band from 700 to 1200 nm is indicative of the unreduced Cr^4+^. b,c) The HRTEM images of the samples annealed at 1050 °C (b) and 1250 °C (c). The insets are the fast Fourier transform (FFT) patterns. d) The TEM image of YCAS:0.04Cr^3+^ ceramics annealed at 1050 °C. The SEM images of YCAS:0.04Cr^3+^ ceramics annealed at 1150 °C (e) and 1250 °C (f). The insets of d–f) are the size distributions. Scale bars: 10 nm (b), 5 nm (c), 50 nm (d), 200 nm (e), and 500 nm (f).

As shown in **Figure** **1**a, the body color of the glass precursor is brownish‐black while the resultant ceramics are green, which is due to the reduction of multivalent chromium to trivalent one. The crystal structure was confirmed by Rietveld refinements of the powder X‐ray diffraction (XRD) patterns (Figure S4 and Tables S1–S3, Supporting Information). Then we conducted high‐resolution transmission electron microscopy (HRTEM) to better understand the microstructure of the ceramic samples annealed at 1050 and 1250 °C, respectively. The HRTEM images (Figure 1b,c) show clear lattice fringes with interplanar spacings corresponding to the garnet structure, and no detectable amorphous phase in both of the samples confirms their full crystallization. The composition of YCAS:Cr^3+^ was determined by the energy‐dispersive X‐ray spectroscopy (EDS) (Table S4, Supporting Information), and STEM‐EDS element mappings (Figure S5, Supporting Information) demonstrate the uniform distribution of the elements of YACS:Cr^3+^ ceramics. Besides, TEM and scanning electron microscope (SEM) images (Figure 1d–f) show that the grain size increases from about 20 nm to submicron as the annealing temperature is elevated from 1050 to 1250 °C owing to the enhanced grain boundary mobility at higher temperature.

### 2.2 Luminescence Properties of YCAS:Cr^3+^ Ceramics

**Figure 2:**
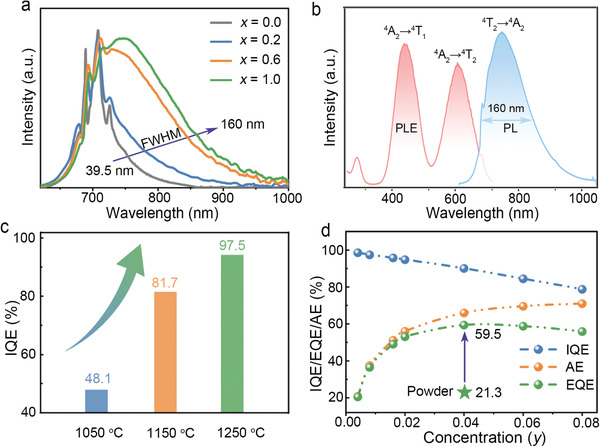
Room‐temperature luminescence properties. a) The normalized emission spectra of Y_3−_ *_x_* Ca*_x_* Al_4.96−_ *_x_* Si*_x_* O_12_:0.04Cr^3+^ (*x* = 0.0, 0.2, 0.6, 1.0) phosphor (*x* = 0.0) or ceramics (*x* = 0.2, 0.6, 1.0). Note that the composition of Y_3_Al_5_O_12_:0.04Cr^3+^ cannot be melted and quenched into glass. b) The excitation and emission spectra of YCAS:0.04Cr^3+^ ceramic (*λ*_ex_ = 440 nm, *λ*_em_ = 760 nm); c) The IQE values of YCAS:0.008Cr^3+^ ceramics annealed at different temperatures. d) The EQE, IQE and AE of YCAS:*y*Cr^3+^ (*y* = 0.004–0.08) ceramics annealed at 1250 °C. The EQE of YCAS:0.04Cr^3+^ phosphor powder is shown for comparison.

Since the small octahedral Al^3+^ site for Cr^3+^ in YAG has strong crystal field on Cr^3+^,^[^ ^21^, ^23^ ^]^ upon violet (430 nm) excitation YAG:Cr^3+^ emits narrowband far‐red light originating from the ^2^E → ^4^A_2_ transition (**Figure** **2**a and Figure S6, Supporting Information). With the cosubstitution of (Y^3+^—Al^3+^) by (Ca^2+^—Si^4+^), the emission band of Cr^3+^ undergoes a gradual redshift from far‐red to NIR range with FWHM increased from 39.5 to 160 nm (Figure S6, Supporting Information), indicating a weakening crystal field imposed on Cr^3+^ according to the Tanabe‐Sugano diagram (Figure S7, Supporting Information).^[^ ^23a^ ^]^ The reduced crystal field can be definitely demonstrated by the calculated spectroscopic parameters that the ratio of crystal field strength parameter (*Dq*) to Racah parameter (*B*) decreases from 2.58 (*x* = 0.2) to 2.40 (*x* = 1.0) (Table S5, Supporting Information). Furthermore, the low‐temperature (77 K) spectra

(Figure S8, Supporting Information) show that a long‐wavelength emission at >750 nm appears after the cosubstitution of Ca^2+^—Si^4+^ (*x* = 0.2, 0.6, 1.0) and the decay curves (Figure S9, Supporting Information) of the peak emission of YCAS:Cr^3+^ (*x* = 1.0) strongly deviate from the single exponential function, implying that the cation cosubstitution gives rise to a new octahedral Al^3+^ site for Cr^3+^ with weak crystal field. It can be attributed to the enlargement of AlO_6_ octahedra owing to the structural distortion caused by Ca^2+^—Si^4+^ cosubstitution.^[^ ^23b^ ^]^ More specifically, the magnitude of tetrahedral shrinkage due to the replacement of larger Al^3+^ by smaller Si^4+^ is larger than that of dodecahedral expansion due to the simultaneous substitution of Ca^2+^ for Y^3+^ (Table S3, Supporting Information), leading to overall shrinkage of the garnet structure (Table S1, Supporting Information); meanwhile, the large shortening of the Si/Al—O bonds pushes the oxygen atoms of the common apex of AlO_6_ octahedra away from the central Al^3+^ and thus leads to the octahedral enlargement. Only when Cr^3+^ occupies a large octahedral site with weak crystal field can it generate broadband NIR emission;^[^ ^23a^ ^]^ accordingly, nearly all of the reported phosphors are gallate/germanate compounds or those containing rare metal ions like Sc^3+^ and In^3+^,^[^ ^8^, ^9^, ^11^, ^12^, ^14^ ^]^ which make them high in cost. By contrast, the case of YCAS:Cr^3+^ implies that it is possible to obtain weak‐field octahedral Al^3+^ sites for generating broadband NIR emission with low cost.

As shown in Figure 2b, YCAS:Cr^3+^ not only has the peak emission wavelength (760 nm) within NIR range and broad emission band (FWHM = 160 nm) due to the ^4^T_2_ → ^4^A_2_ transition, but also possesses broad excitation band (^4^A_2_ → ^4^T_1_ transition) peaking at 440 nm that matches well with the commercialized blue LEDs. Translucent YCAS:Cr^3+^ ceramics are thus deemed the optimal converters for broadband NIR pc‐LEDs among the designed solid solution ceramics with single garnet phase. Although a temperature of 1050 °C is sufficient to induce the full crystallization, annealing at a little higher temperature (1250 °C) is highly preferred regarding the luminescence properties. As one of the key parameters for light‐converting materials, the IQE of YCAS:0.008Cr^3+^ can be significantly improved from 48.1% to as high as 97.5% as the annealing temperature is increased from 1050 to 1250 °C (Figure 2c). One reason is that larger grain size at higher annealing temperature ensures better crystallinity and thus fewer surface defects which usually act as luminescence killers.^[^ ^21c^ ^]^ Besides, the reduction of Cr^4+^ to Cr^3+^ can be completed only at higher temperatures, as indicated by the disappearance of the characteristic absorption band (Figure 1a) of Cr^4+^ around 1000 nm.^[^ ^9^, ^21^ ^]^ The IQE of YCAS:*y*Cr^3+^ ceramics annealed at 1250 °C remains to be 90.1% at the optimum Cr^3+^ doping concentration (*y* = 0.04) (Figure 2d). Remarkably, the AE of translucent YCAS:0.04Cr^3+^ ceramic reaches up to 66.0%, leading to a record EQE of 59.5% for broadband NIR emission (see Table S6, Supporting Information, for detailed comparison). Note that the EQE (IQE) of the powder‐type counterpart is only 21.3% (71.4%) (Figure 2d and Table S7, Supporting Information).

**Figure 3:**
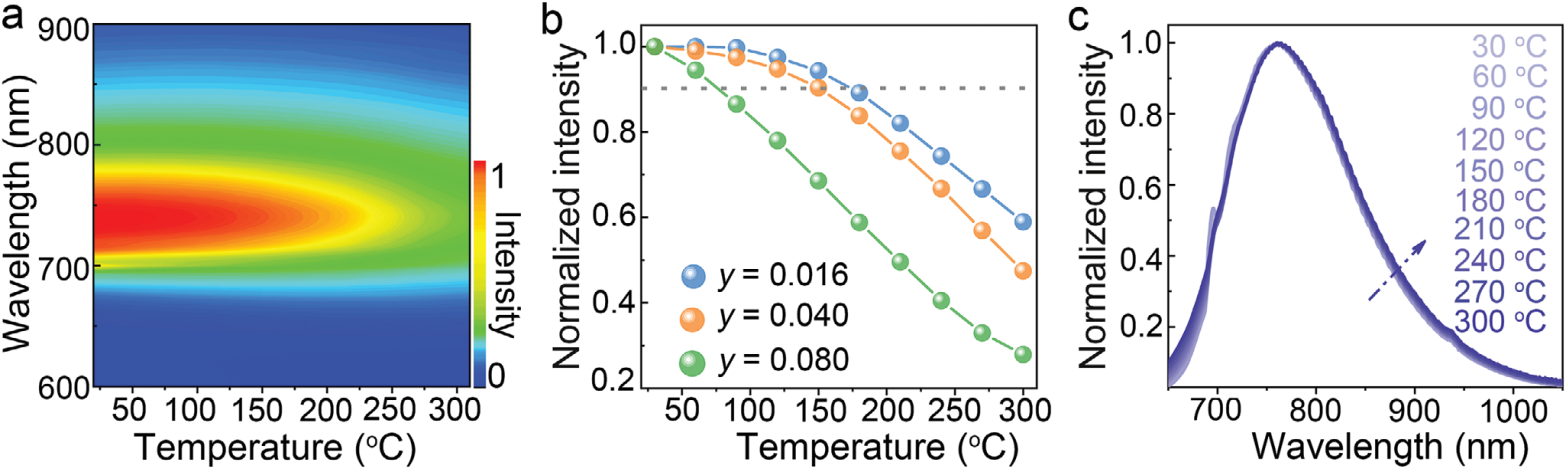
High‐temperature luminescence properties. a) A contour plot of the emission spectra (*y*‐axis) and temperature (*x*‐axis) showing the color stability with increasing temperature for YCAS:0.04Cr^3+^ (*λ*_ex_ = 440 nm) annealed at 1250 °C. b) Integrated emission intensities of YCAS:*y*Cr^3+^ ceramics (*y* = 0.016, 0.04, and 0.08) annealed at 1250 °C as a function of the temperature. Note that the values are normalized by that at 30 °C. c) Normalized emission spectra of YCAS:0.04Cr^3+^ ceramics annealed at 1250 °C at different temperatures.

The formidable problem of pc‐LEDs is the performance deterioration of the converters during long‐term high‐temperature (>150 °C) operation for the heat generation in light down‐conversion, which is much worse for the case of broadband NIR pc‐LEDs. The IQE loss of phosphors due to thermal quenching will further push up the operating temperature. One mitigation strategy is to adopt luminescence bulk materials with high thermal conductivity like translucent YCAS:Cr^3+^ ceramics developed here (≈3.5 W m^−1^ K^−1^)^[^ ^26^ ^]^ to circumvent the organic encapsulation. From the viewpoint of luminescence centers/particles, it demands thermally stable emission in intensity as well as profile. As shown by the temperature‐dependent emission spectra (**Figure** **3**a and Figure S10, Supporting Information), the emission intensity decreases with temperature owing to the thermally enhanced non‐radiative rate (Figure S11, Supporting Information), and it becomes more obvious for higher Cr^3+^ concentrations (Figure 3b and Figure S12, Supporting Information) due to the thermally activated concentration quenching.^[^ ^16^ ^]^ Importantly, 90.6% of the emission intensity of YCAS:0.04Cr^3+^ ceramic is still retained as the temperature is elevated from 30 to 150 °C. Moreover, both the peak and the width exhibit very small variation with temperature (Figure 3c). The excellent thermal stability of YCAS:Cr^3+^ possibly benefits from the 3D connected structure of garnet crystals and the highly charged cations (Al^3+^/Si^4+^) with small radii enabling the higher rigidity of aluminosilicate garnets than gallate/germanate ones.^[^ ^16c^ ^]^ To the best of our knowledge, this is the first time to simultaneously achieve high EQE (IQE) and excellent thermal stability for broadband NIR emission (Table S6), highlighting the superiority of glass crystallization over traditional powder sintering (or high‐temperature solid‐state reaction) in the synthesis of luminescence materials, especially bulk luminescence ceramics.

### 2.3 High‐Performance Broadband NIR pc‐LEDs

**Figure 4:**
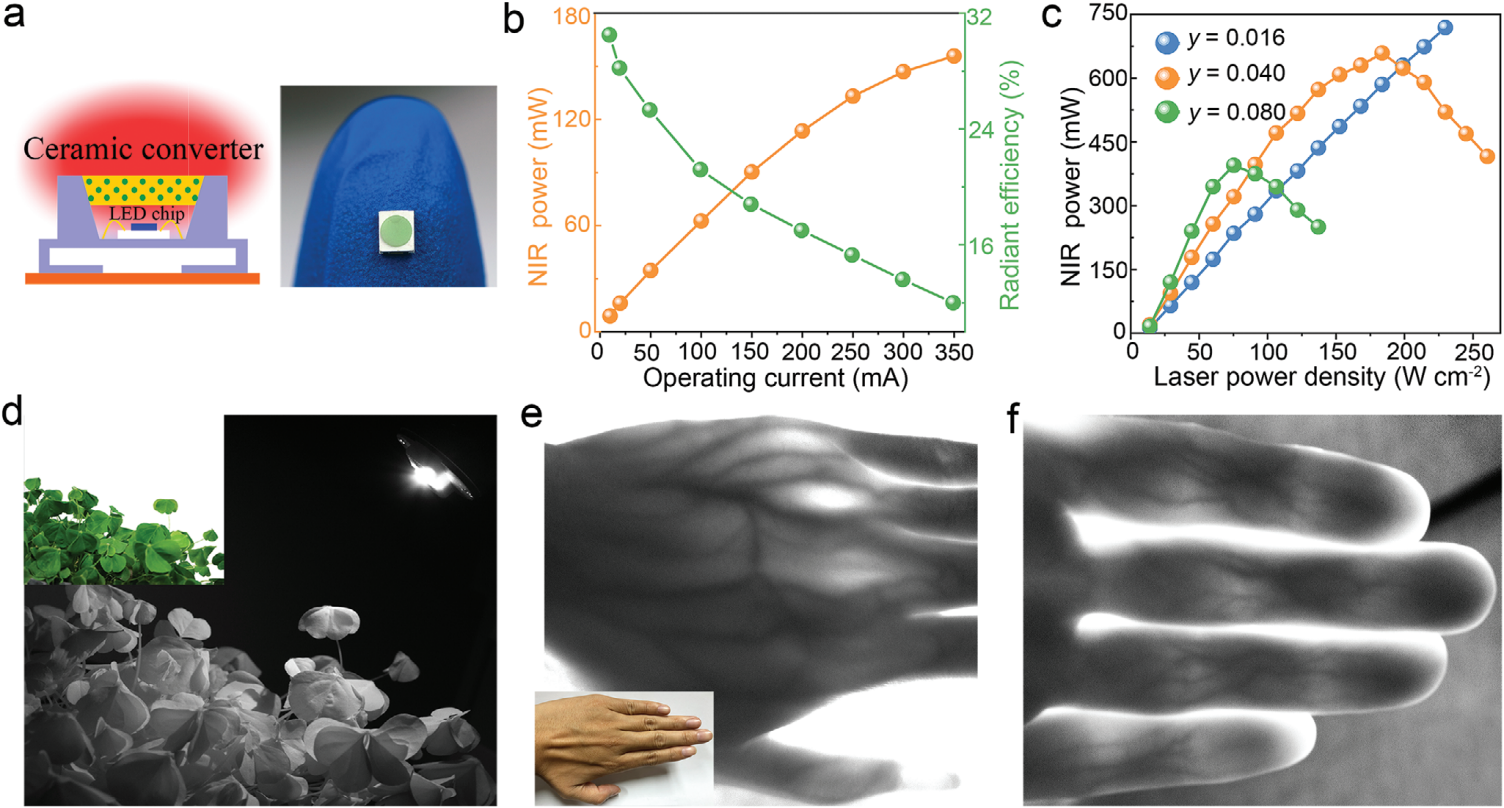
Demonstration of all‐inorganic NIR pc‐LEDs. a) The illustration and photograph of the as‐prepared NIR pc‐LED device by combining translucent YCAS:Cr^3+^ ceramics (0.4 mm in thickness) with a 450 nm LED chip (3 × 3 mm, 1 W). Silicone is used to just seal the chink. b) NIR output power and photoelectric efficiency of the pc‐LED as a function of driving current. The NIR photoelectric efficiency is defined as the percentage of the output power of NIR light (650–1100 nm) to the input electric power of pc‐LED. c) NIR light output power of YCAS:Cr^3+^ ceramics with different Cr^3+^ concentrations (*y*) as a function of the power density of the incident 450 nm laser. d) Photo of the clover lighted by the as‐fabricated NIR pc‐LED in the darkness, which is taken by a NIR camera. The inset is the photo of the clover under day light. e,f) NIR transilluminated photos of the fingers and the palm to clearly show the veins therein. The inset is the photo of the corresponding hand under day light.

The shortcomings of “phosphor‐in‐silicone (PiS)”, the routine packaging method in the field of pc‐LEDs, are exacerbated in the case of blue‐to‐NIR conversion. For instance, in the previous works, the NIR light output power started to decrease at a driving current far below the maximum value of blue LED (1 W) chips.^[^ ^9^, ^14^ ^]^ As illustrated in **Figure** **4**a, we here fabricated all‐inorganic NIR pc‐LED prototypes by directly implanting the translucent YCAS:Cr^3+^ ceramics into the groove of tiny 450 nm LED chips (1 W) and just sealing the chink with silicone. For comparison, we also fabricated traditional NIR pc‐LEDs using YCAS:Cr^3+^ phosphor powders packaged by cured silicone. The NIR photoelectric efficiency is as high as 30.5% at 10 mA (2.66 V) for the pc‐LED device based on YCAS:0.04Cr^3+^ ceramics (Figure 4b), which is about 2.5 times higher than that of its PiS counterpart (12.4%@10mA); an NIR output power of 62.6 mW can be also obtained at 100 mA (3 V) with the efficiency decreased to 21.2% due to the “efficiency droop” of the LED chip (Figure S13, Supporting Information); these electroluminescence properties are much better than those of the previously reported ones (Table S8, Supporting Information).^[^ ^9^, ^10^, ^14^ ^]^ Notably, the NIR output power of the all‐inorganic pc‐LEDs increases monotonously with the input current within the rated current (350 mA) of blue LED chips (Figure 4b and Figure S14, Supporting Information), whereas the saturation can be clearly observed in the case of YCAS:0.08Cr^3+^ powders (Figure S15, Supporting Information).

To further evaluate their application potential in high‐power scenarios, we adopted a power‐tunable 450 nm laser diode (LD) as the excitation source to demonstrate laser‐driven broadband NIR light sources in a reflective mode (Figure S16, Supporting Information). As shown in Figure 4c, YCAS:0.04Cr^3+^ ceramic shows no luminescence saturation before the laser power density was turned up to 184 W cm^−2^, suggesting the excellent tolerance under high‐density blue light irradiation. The saturation threshold is reduced when increasing the Cr^3+^ concentration, which is in accordance with the trend of the IQE and the thermal stability. Using the as‐fabricated NIR pc‐LED as the non‐visible light source, we can take a clear black‐and‐white photograph for the clover in the darkness by a NIR camera (Figure 4d). Besides, the veins in the fingers, as well as the palm with a thickness of ≈2.0 cm, can be well‐identified because of the good penetration of NIR light and the difference in light absorption of hand tissues (Figure 4e,f). These proof‐of‐concept demonstrations reveal the advantages of YCAS:Cr^3+^ ceramics in generating high‐power and high‐efficiency broadband NIR light and manifest their great potential in the field of NIR applications, such as night vision, biological imaging, and NIR spectroscopy. The volunteer was informed about the experiment before participating in the study and gave its written consent.

## 3 Discussion and Outlook

Unlike powder compacts, densified ceramics with weak light scattering allows a much longer path length of the incident light beam (Figure S17, Supporting Information), thereby increasing the probability of being absorbed by the activators with a given concentration.^[^ ^17^, ^18^ ^]^ Considering high thermal conductivity and excellent physical and chemical stabilities, luminescence transparent/translucent ceramics are expected to be a “three birds with one stone” strategy for improving the AE of Cr^3+^ without compromising the IQE and the thermal stability. Except for garnet phosphors,^[^ ^9d,g^ ^]^ all the Cr^3+^ activated NIR phosphors with broadband emission are not optically isotropic, and the oxides of Ga, Ge, and In with high vapor pressure are prone to volatilization at high temperature and high vacuum,^[^ ^27^ ^]^ which make broadband NIR‐emitting ceramics inaccessible to traditional sintering methods. Several researchers,^[^ ^23^, ^25^, ^28^ ^]^ as well as us, attempted to synthesize phosphor powders or bulk ceramics based on YCAS garnet by traditional sintering methods, but all failed to obtain the single phase (Figure S18, Supporting Information), leading to poor optical properties (Table S7, Supporting Information).

Intriguingly, we show here that single‐phase YCAS can be easily obtained via crystallization from the corresponding glass precursor, providing us a new route to developing highly efficient NIR‐emitting ceramics at low temperature and atmospheric pressure. We note that despite the high transparency of YCAS glass, YCAS ceramics have a relatively low transparency (28% at 450 nm for the sample annealed at 1250 °C) (Figure S19, Supporting Information). The existence of pores inside the bulk YCAS ceramics (Figure S20, Supporting Information) is responsible for the transparency reduction, since YCAS crystal has cubic symmetry to ensure the optical isotropy. These pores stem from the strong contraction during glass crystallization due to the large difference in density between YCAS glass (3.61 g cm^−3^) and YCAS crystal (4.08 g cm^−3^).^[^ ^19^ ^]^ Therefore, more attention should be paid to eliminating the residual pores for controllable light scattering in the future, such as optimizing the annealing schedule and using appropriate sintering aids.^[^ ^18^, ^29^ ^]^

For practical applications, especially in NIR spectroscopy as well as solar simulators, the emission of NIR pc‐LEDs should be further extended to include the short‐wave NIR light of 950–1100 nm within the response range of silicon detectors. However, Cr^3+^ cannot generate efficient and stable emission in this spectral range.^[^ ^9^, ^12^ ^]^ Instead, trivalent ytterbium ion (Yb^3+^) is an excellent short‐wave NIR emitter, and its blue light excitation can be realized via the sensitization of Cr^3+^.^[^ ^30^ ^]^ Thanks to the suitable Y^3+^ sites for Yb^3+^ occupation, the short‐wave NIR emission can be enriched by introducing Yb^3+^ into YCAS:Cr^3+^ (Figure S21, Supporting Information). Moreover, YCAS:Cr^3+^,Yb^3+^ have ultrahigh IQE (>95%) and nearly zero thermal quenching (Figure S22, Supporting Information), which is attributed to efficient energy transfer from Cr^3+^ to Yb^3+^.^[^ ^30^ ^]^ Finally, we also anticipated that it is possible, though very challenging, for glass crystallization to achieve efficient Cr^3+^ activated bulk materials with longer peak emission wavelength (>800 nm) as well as extra‐broad emission band, through rational design of the glass composition and fine control of the crystalline phases.^[^ ^19^, ^20^, ^21^ ^]^

In summary, we have successfully developed a broadband NIR‐emitting translucent ceramic via a facile preparation method based on pressureless glass crystallization. Taking the advantages of glass crystallization over traditional powder sintering and luminescence ceramics over phosphor powders, we simultaneously achieved high EQE (IQE), high thermal stability, easy fabrication, and low cost for broadband NIR converters. As such, we demonstrated all‐inorganic broadband NIR pc‐LEDs with excellent optical performance, which may find a wide range of optical applications including night vision, miniature NIR spectrometers, and LED solar simulators. We are also convinced that glass crystallization is a cost‐effective and promising approach to developing efficient luminescence ceramics for high‐power non‐visible light sources.

## 4 Experimental Section

### Materials and Preparations

Y_3−_ *_x_*_−_ *_z_* Ca*_x_* Al_5−_ *_x_*_−_ *_y_* Si*_x_* O_12_:*y*Cr^3+^,*z*Yb^3+^ (*x* = 0.2–1.4, *y* = 0.004–0.08, *z* = 0.01–0.03) translucent ceramics were synthesized via a pressureless glass crystallization. The high‐purity raw materials of Y_2_O_3_ (99.999%), CaCO_3_ (99.999%), Al_2_O_3_ (99.999%), SiO_2_ (99.999%), Cr_2_O_3_ (99.99%), Yb_2_O_3_ (99.999%) were stoichiometrically weighed and mixed in an agate mortar for 30 min. Then the glass beads with a diameter of 4–5 mm were obtained by the aerodynamic levitation method (Figure S1, Supporting Information). The detailed processes can be found in the previous work (Ref. [21b]). The polished ceramic plates with thicknesses of 0.2–1.0 mm were obtained by annealing the glass samples in a tube furnace for 10 h at temperatures of 1050 to 1250 °C in 95% N_2_+5% H_2_ atmosphere.

### Characterizations

Differential thermal analysis (DTA) of glass sample was measured on a CRY‐Z differential thermal analyzer at a heating rate of 10 °C min^−1^. XRD patterns were obtained on a Rigaku D diffractometer (Cu K*α* radiation, *λ* = 1.54 Å) with a scanning range of 2*θ* = 10–120° and a scanning rate of 10° per min. The Rietveld refinement analysis was performed by using the FullProf program. The surface morphology and compositions of the samples were characterized by a transmission electron microscope (Talos F200s, Thermo Scientific) operated at 200 kV with an energy‐dispersive spectrometer (EDS), and a field‐emission scanning electron microscopy (SEM) (SU‐8010, Hitachi, Japan) at the operating voltage of 30.0 kV, where a dual‐beam FIB microscope (Strata 400S, FEI) was used to thin the samples. A UV–Vis–NIR spectrophotometer (UH5700, Hitachi) was applied to record the total transmittance of samples. The luminescence properties including emission and excitation spectra, decay curves, and temperature‐dependent emission spectra were measured by using a fluorescence spectrometer (FLS920P, Edinburgh Instrument Ltd.), where the temperature was finely controlled by a high‐temperature controller (TAP‐02, Orient KOJI) or a liquid nitrogen cryostat (CRYO‐77, Orient KOJI). The IQE and the absorption were recorded by a UV–NIR absolute photoluminescence quantum yield spectrometer (Quantaurus‐QY Plus C13534‐12, Hamamatsu Photonics) and the error is about 1% for three times independent measurements. The density of bulk glass and ceramics was measured using the Archimedes method with absolute ethyl alcohol as the immersion fluid.

### Device Fabrication and Performance Measurements

The NIR pc‐LEDs were fabricated by directly covering LED chips (1 W, 450 nm) with YCAS:Cr^3+^ ceramics annealing at 1250 °C for 10 h, and then sealed by highly transparent silicone (Figure 4a). The electroluminescence properties of pc‐LED devices including the electroluminescence spectra and photoelectric efficiency were characterized on an integrated photoelectric measurement system (LHS‐1000, EVERFINE), which was equipped with an array spectrophotometer (350–1100 nm, HAAS‐2000) and an integrating sphere (SPEKTRON R98, Φ 50 cm). A 450 nm LD (LSR450CP‐15W‐FC, LASEVER, Ningbo) with controllable laser power was used as the excitation source to evaluate the performance of the samples at high‐density radiation. The spot area was fixed at 3.14 mm^−2^. All the photographs were taken by a digital camera (Canon 750D) or NIR camera (MVCA050‐20GN, Hikvision, China).

## Conflict of Interest

G.Z., W.X., and J.Q. are inventors on a Chinese patent application (202110297549.1) related to this work. All other authors declare no competing interests.

## Author Contributions

W.X., G.Z., and J.Q. conceived the idea and designed the experiments. J.Q. and W.X. supervised the project. G.Z., J.W., and W.X. performed the experiments. W.X. and G.Z. analyzed the data, and all the authors discussed the results. W.X. and G.Z. wrote the manuscript with input from others.

## Supporting information

Supporting Information

## Data Availability

The data that support the findings of this study are available from the corresponding author upon reasonable request.
