# Supplementary material for: Glass‐Crystallized Luminescence Translucent Ceramics toward High‐Performance Broadband NIR LEDs
Source: Adv Sci (Weinh). 2022 Jan 24;9(8):2105713. doi: 10.1002/advs.202105713 (PMC8922114; doi:10.1002/advs.202105713)
Supplement: Supplementary file 1 — Supporting Information [file ADVS-9-2105713-s001.pdf]

ADVANCED SCIENCE
Open Access

Supporting Information

for *Adv. Sci.*, DOI 10.1002/advs.202105713

Glass-Crystallized Luminescence Translucent Ceramics toward High-Performance Broadband NIR LEDs

*Guojun Zheng, Wenge Xiao**, Jianhong Wu, Xiaofeng Liu, Hirokazu Masai and Jianrong Qiu***

WILEY-VCH

ADVANCED SCIENCE
Open Access

Supporting Information

for *Adv. Sci.*, DOI: 10.1002/advs.202105713

Glass-crystallized Luminescence Translucent Ceramics toward High-performance Broadband NIR LEDs

Guojun Zheng, Wenge Xiao, Jianhong Wu, Xiaofeng Liu, Hirokazu Masai and Jianrong Qiu**

Supporting Information

Glass-crystallized luminescence translucent ceramics toward high-performance broadband NIR LEDs

Guojun Zheng, Wenge Xiao, Jianhong Wu, Xiaofeng Liu, Hirokazu Masai and Jianrong Qiu**

Dr. G. Zheng, W. Xiao, J. Wu, Prof. J. Qiu
State Key Lab of Modern Optical Instrumentation, College of Optical Science and Engineering, Zhejiang University, Hangzhou 310027 (P. R. China)
E-mail: wengsee@163.com, wengsee@zju.edu.cn (W. X.); qjr@zju.edu.cn (J. Q.)
Prof. X. Liu
School of Materials Science and Engineering, Zhejiang University, Hangzhou 310027 (P. R. China)
Prof. H. Masai
National Institute of Advanced Industrial Science and Technology, Osaka 563-8577, Japan
Prof. J. Qiu
CAS Center for Excellence in Ultra-intense Laser Science, Shanghai Institute of Optics and Fine Mechanics, Chinese Academy of Sciences, Shanghai, 201800 (P. R. China)

This PDF file includes:

Figs. S1 to S22

Tables S1 to S8

References (S1 to S16)

Supplementary Figures

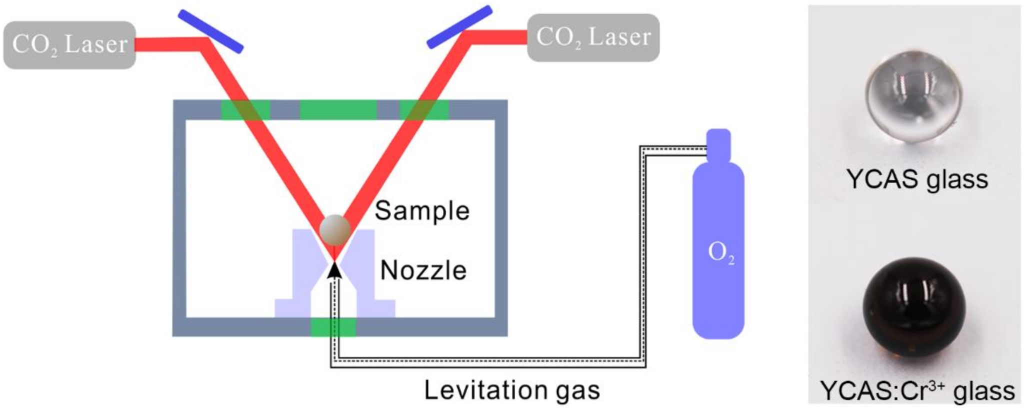

Figure S1 Schematic of an aerodynamic levitation system equipped with two CO_2 lasers for heating. On the right are the as-prepared glass beads.

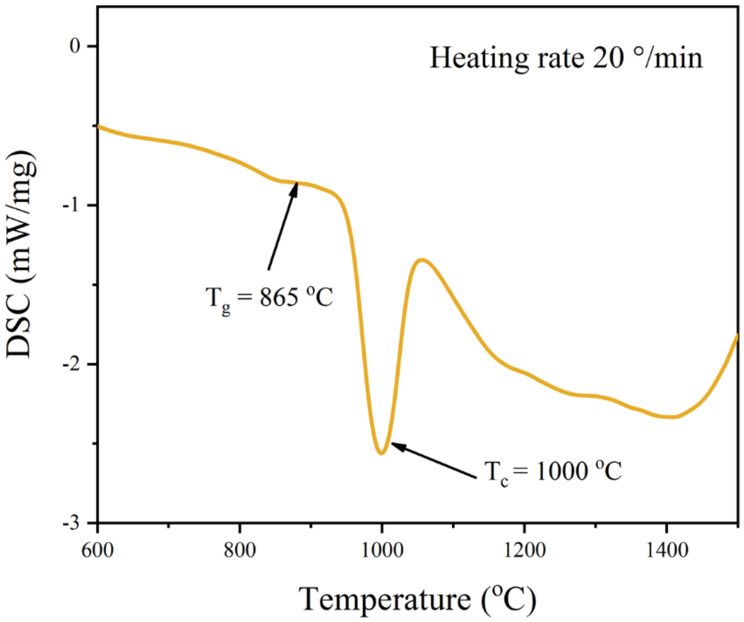

Figure S2 DSC curve of $Y_2CaAl_4SiO_{12}$ glass. It shows only one exothermic peak around 1000 °C, corresponding to the crystallization of garnet phase.

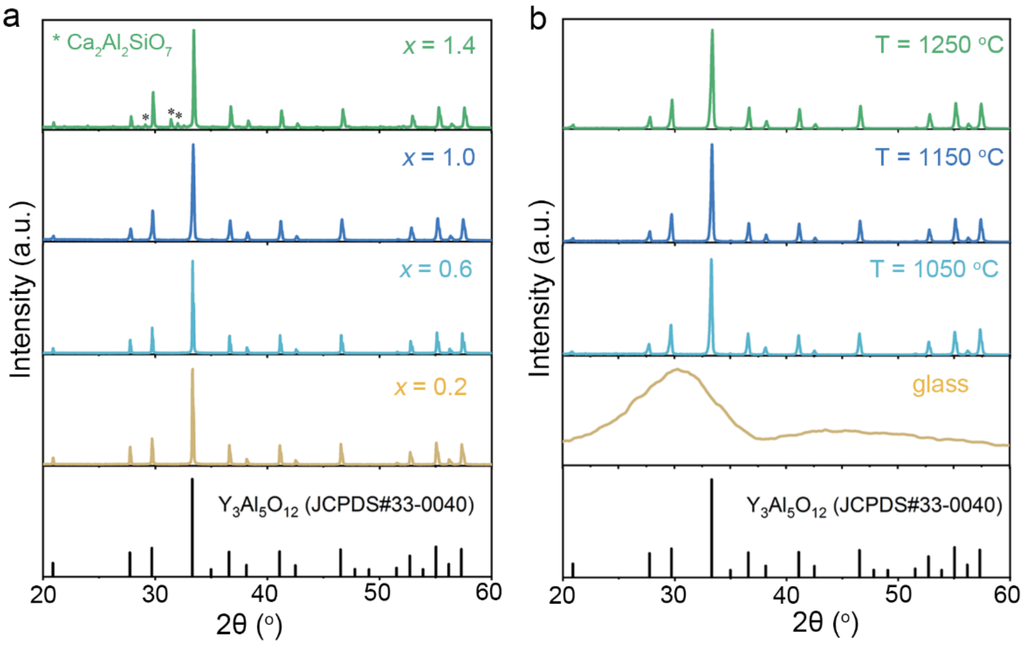

Figure S3 (**a**) The XRD patterns of $Y_{3-x}Ca_xAl_{5-x}Si_xO_{12}$ (x = 0.2–1.4) ceramics annealed at 1250 °C for 10 h. The standard card of $Y_3Al_5O_{12}$ (JCPDS#33-0040) is shown for comparison. Note that a second phase ($Ca_2Al_2SiO_7$) appears at x = 1.4. (**b**) The XRD patterns of $Y_2CaAl_4SiO_{12}$ glass and ceramics annealed at different temperatures for 10h.

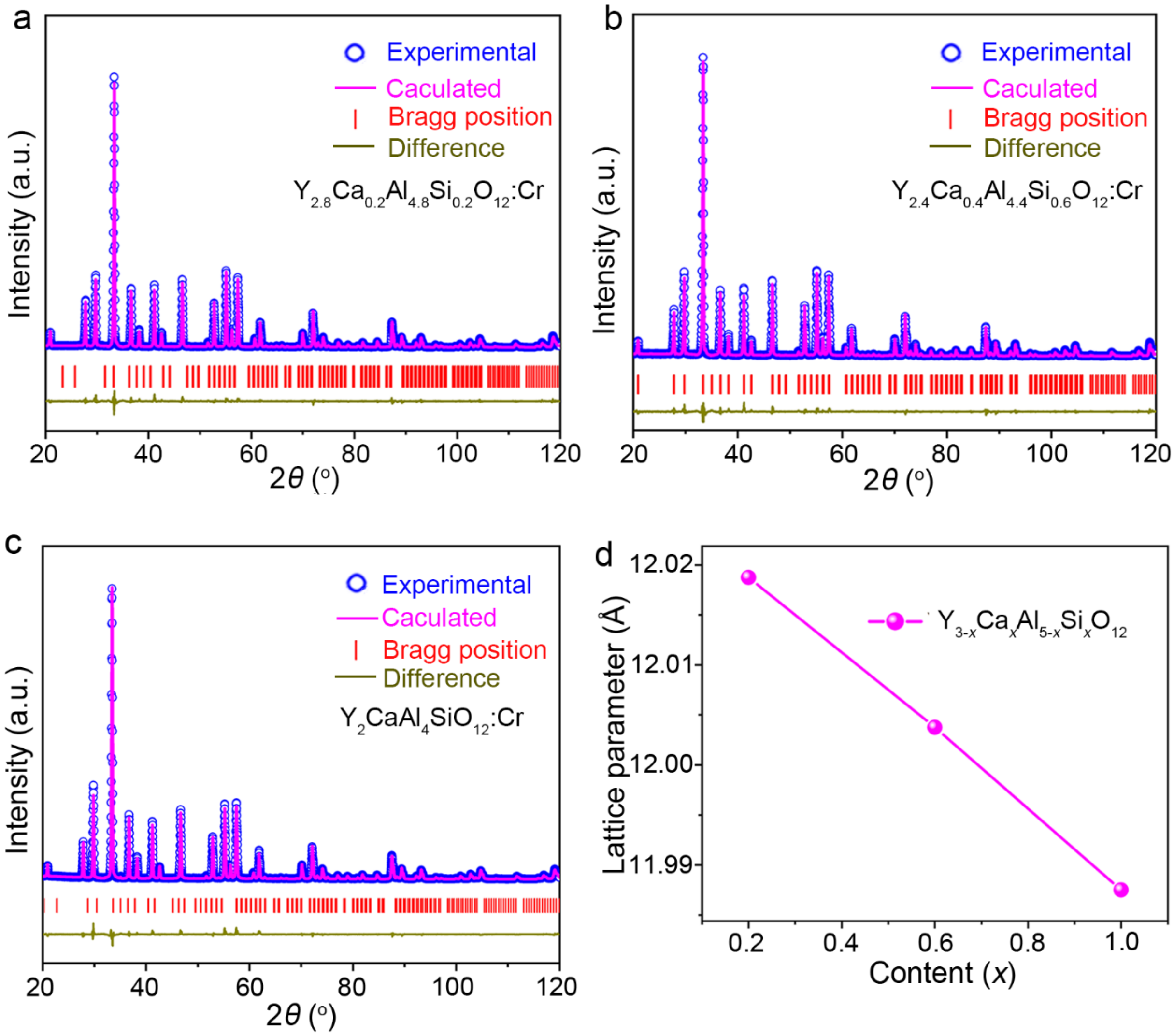

Figure S4 Rietveld refinement of XRD patterns of $Y_{3-x}Ca_xAl_{5-x}Si_xO_{12}$ with $x = 0.2$ (**a**); $x = 0.6$ (**b**); $x = 1.0$ (**c**). (**d**) The cell parameters of $Y_{3-x}Ca_xAl_{5-x}Si_xO_{12}$. Their linear evolutions confirm the formation of the solid solution garnets.

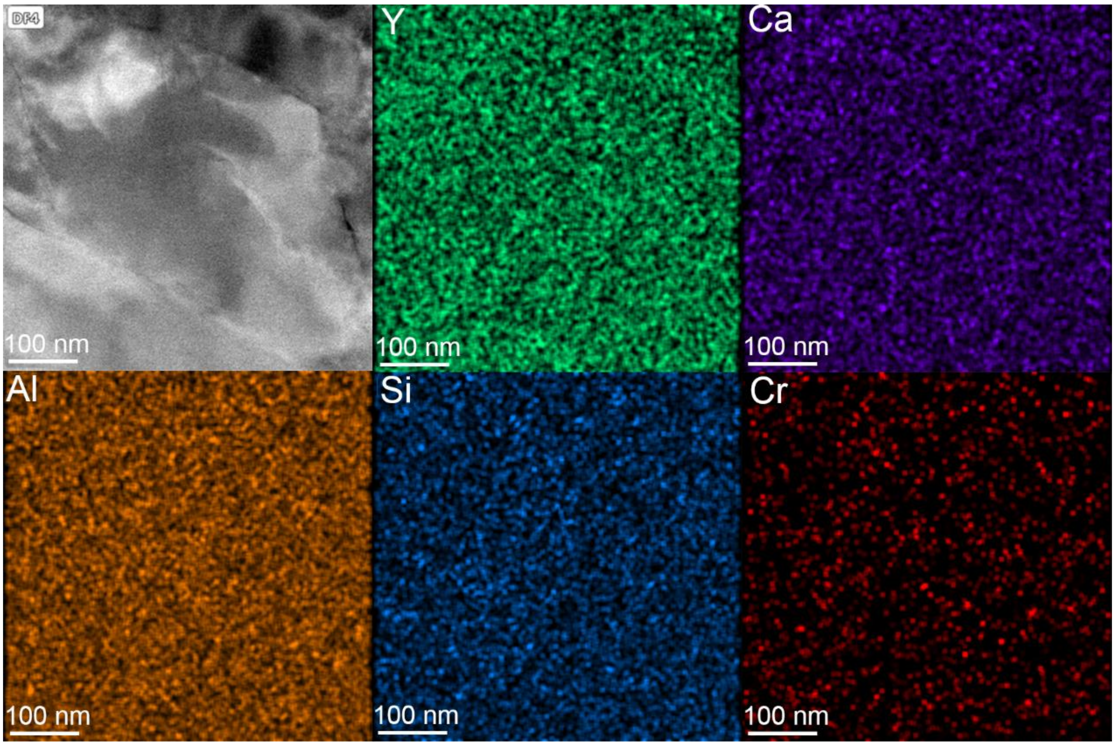

Figure S5 STEM-EDS elemental mappings of YCAS:0.08Cr^{3+}.

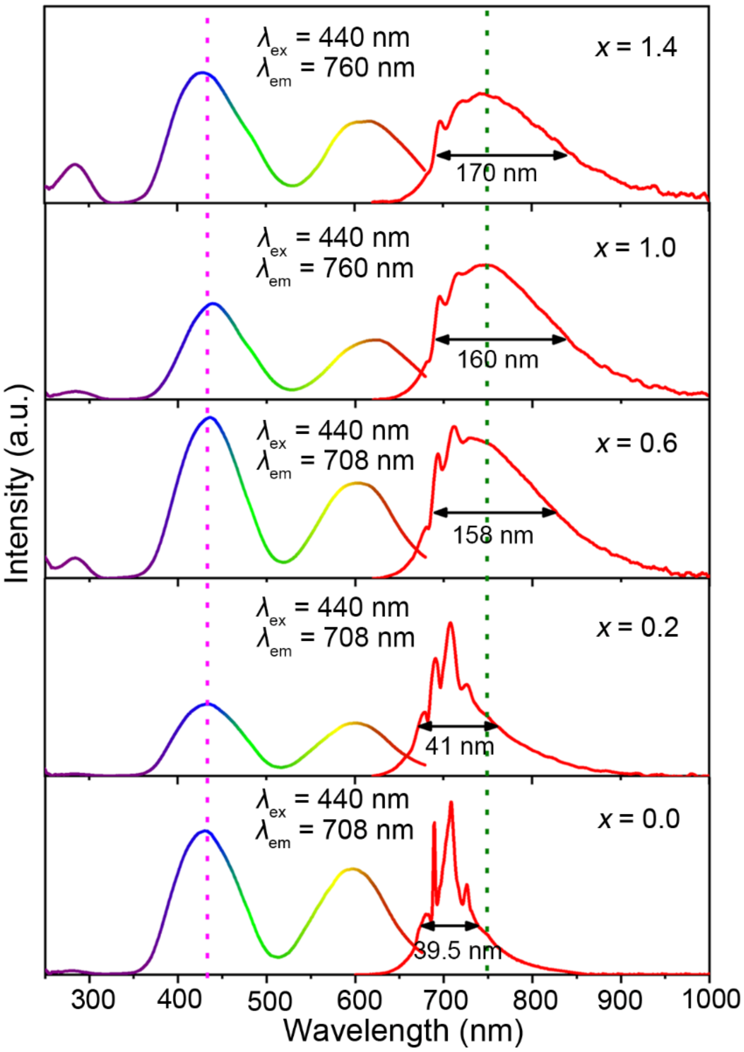

Figure S6 The excitation and emission spectra of $Y_{3-x}Ca_xAl_{4.96-x}Si_xO_{12}$:0.04$Cr^{3+}$ (x = 0.0, 0.2, 0.6, 1.0, 1.4) phosphor (x = 0.0) or ceramics (x = 0.2, 0.6, 1.0, 1.4). Note that the composition of $Y_3Al_5O_{12}$:0.04Cr^{3+} cannot be melted and quenched into glass, and thus we prepared the powder-type sample for measurement.

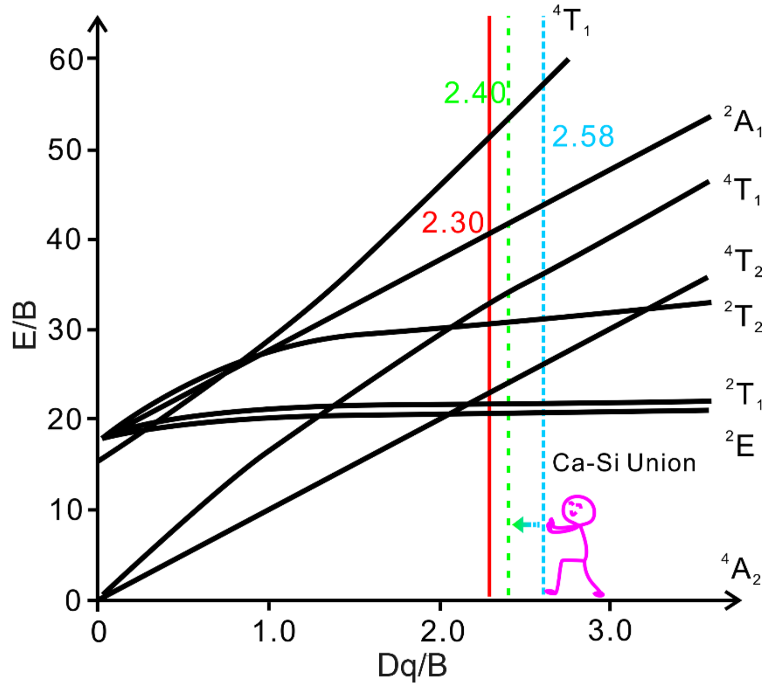

Figure S7 Tanabe-Sugano energy level diagram for Cr^{3+} in the octahedral site with *Dq*/*B* decreased from 2.58 to 2.40 due to the co-substitution of Ca-Si in YAG.

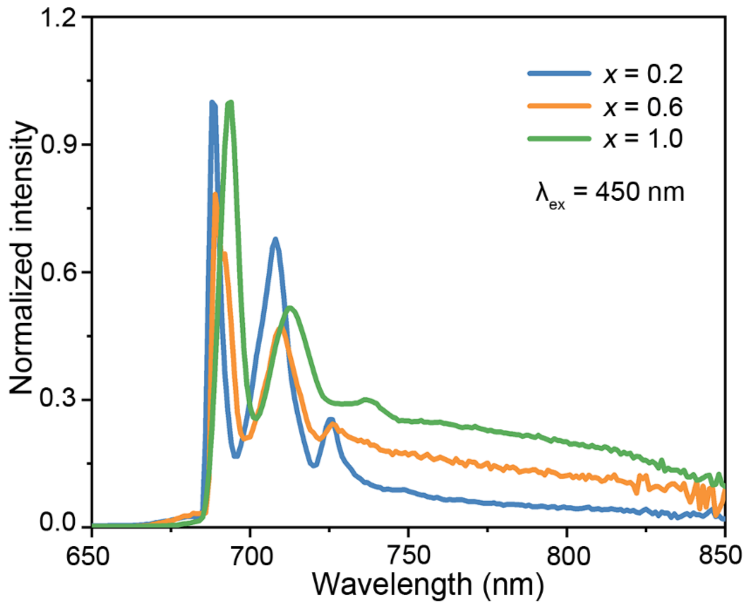

Figure S8 Low-temperature (77 K) spectra of $Y_{3-x}Ca_xAl_{4.96-x}Si_xO_{12}$:$0.04Cr^{3+}$ (x = 0.2, 0.6, 1.0).

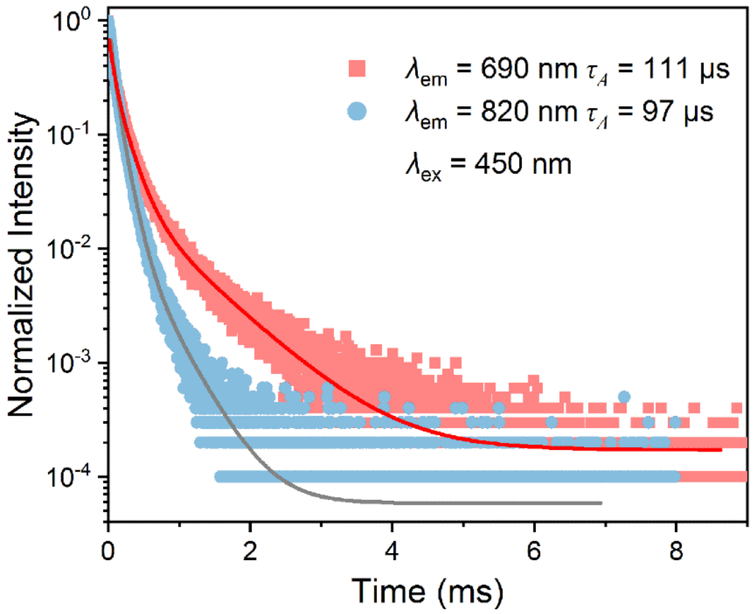

Figure S9 The decay curves of YCAS:0.04Cr^{3+} monitoring at 690 and 800 nm, respectively, under 450 nm excitation. The decay curves can be well fitted by triple-exponential decay function:

$$I(t) = I_0 + A_1 e^{-t/\tau 1} + A_2 e^{-t/\tau 2} + A_3 e^{-t/\tau 3}$$

where *I(t)* is intensity at a given time *t*, and A_1, A_2 and A_3 are constants. The average lifetimes (τ_A) were calculated to be 111 (τ1 = 70 μs, τ2 = 180 μs, τ3 = 755 μs) and 97 μs (τ1 = 8 μs, τ2 = 120 μs, τ3 = 585 μs) for the monitoring wavelength of 690 nm and 820 nm, respectively.

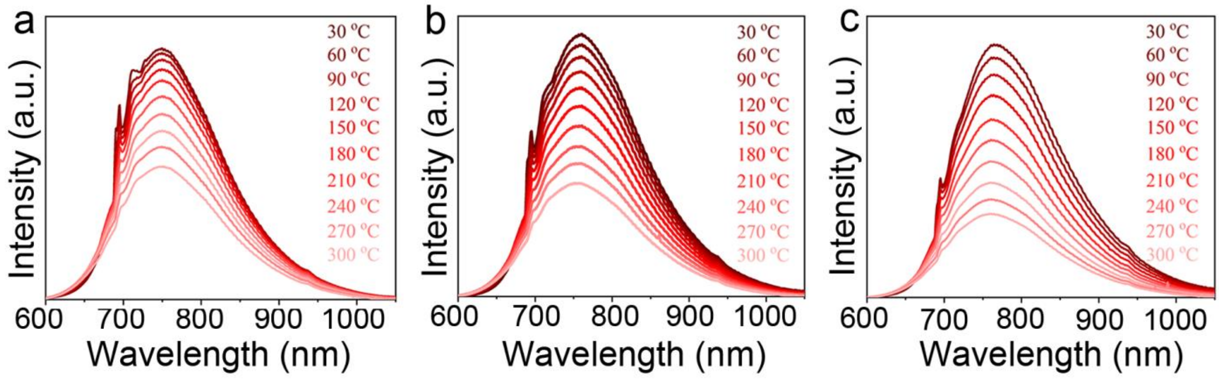

Figure S10 Temperature-dependent spectra of YCAS:0.016Cr^{3+} (**a**), YCAS:0.04Cr^{3+} (**b**), YCAS:0.08Cr^{3+} (**c**).

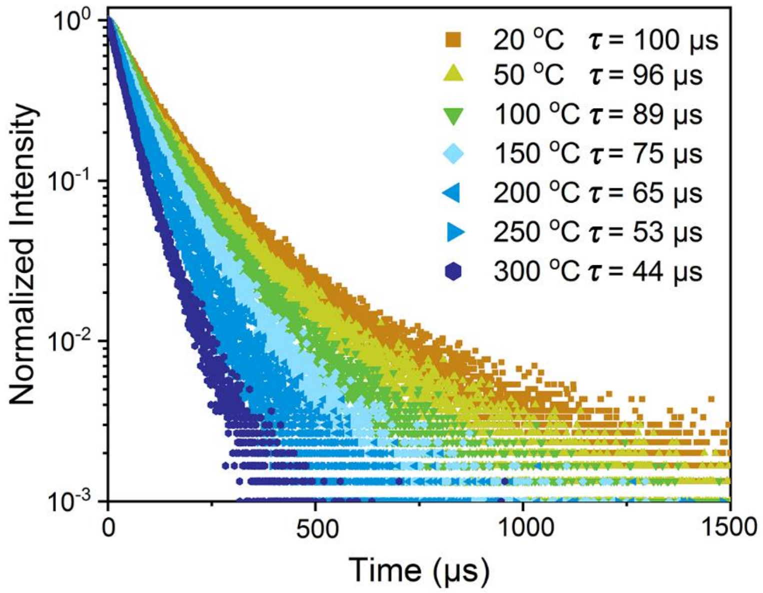

Figure S11 Temperature-dependent decay curves of YCAS:0.04Cr^{3+} (λ_{ex} = 450 nm, λ_{em} = 760 nm).

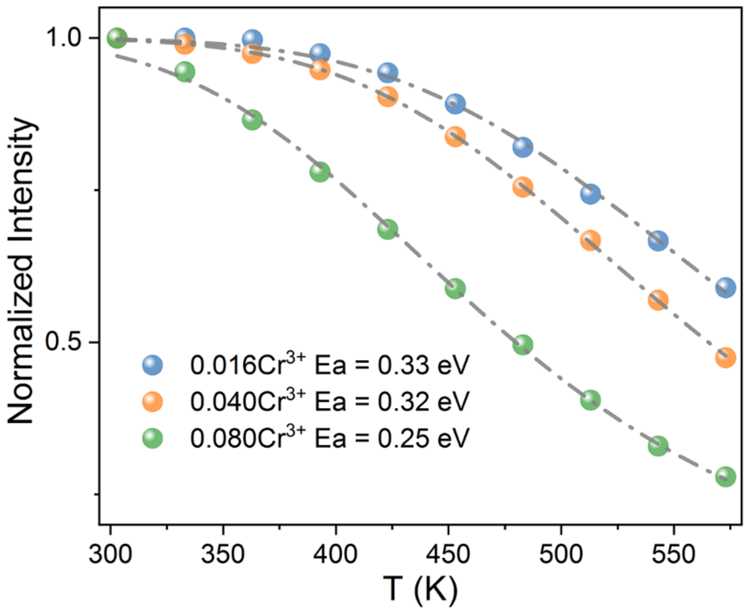

Figure S12 Data fitting using the Arrhenius equation for YCAS:*y*Cr^{3+} ceramics (*y* =0.016, 0.04, and 0.08) annealed at 1250 ^{o}C. The Arrhenius equation is:

$$I(T) = \frac{I_0}{1 + Aexp(-Ea/kT)}$$

where I_0 is the initial integrated PL intensity, *I(T)* is the integrated PL intensity at a given temperature, *A* is a constant, *k* is the Boltzmann constant (8.617×10^{-5} eV K^{-1}) and *Ea* is the activation energy for thermal quenching.

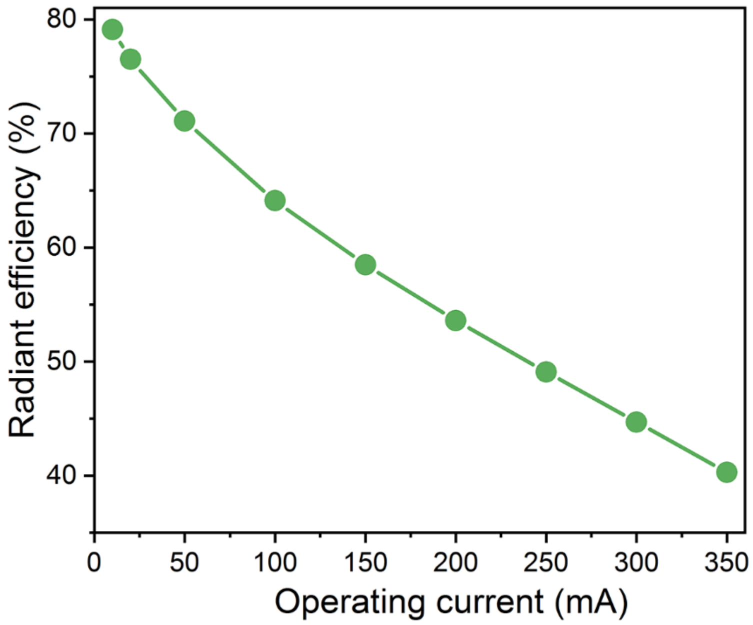

Figure S13 The photoelectric efficiency of 450 nm LED chips used here as a function of driving current.

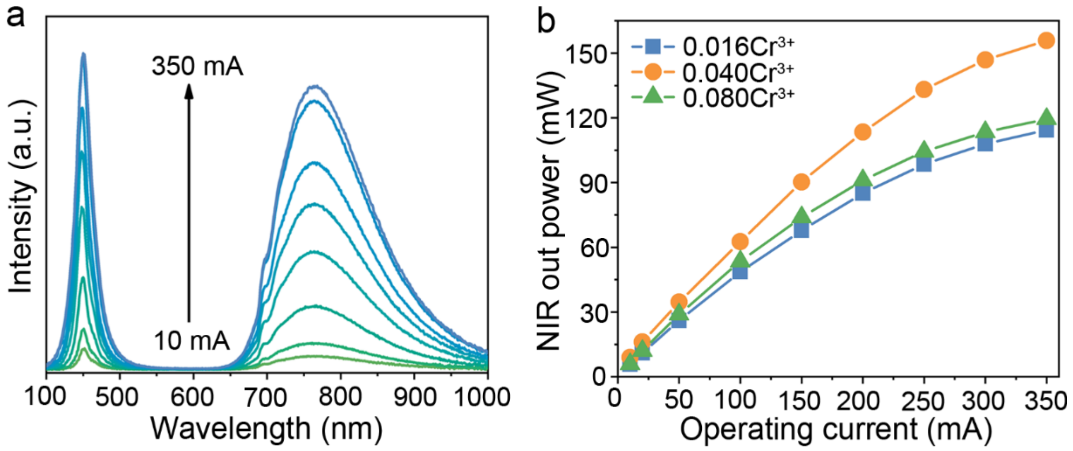

Figure S14 (**a**) The electroluminescence spectra of the NIR pc-LED device based on YCAS:0.04Cr^{3+} ceramics under different operating currents. (**b**) The NIR output power of the as-fabricated pc-LED devices based on YCAS:*y*Cr^{3+} ceramics as a function of driving current for the 450 nm LED chip.

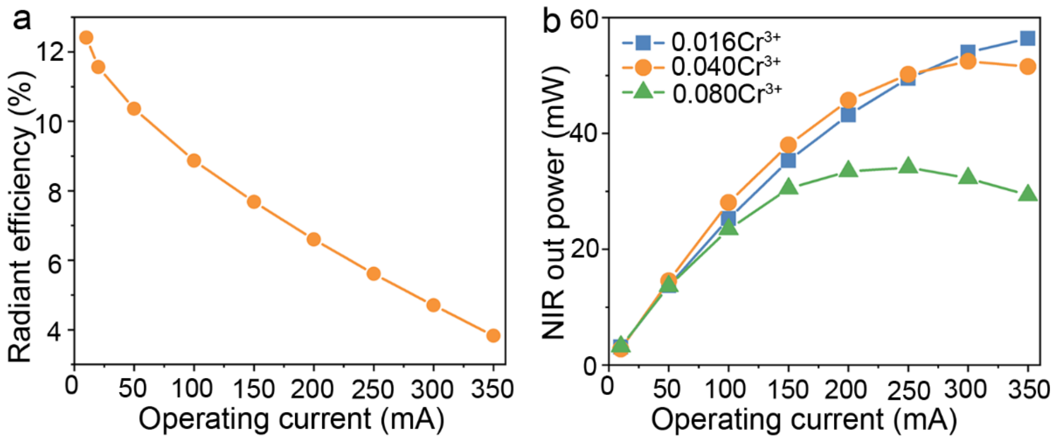

Figure S15 (**a**) The NIR photoelectric efficiency of the pc-LED fabricated by YCAS:0.04Cr^{3+} phosphor powder as a function of driving current. (**b**) The NIR output power of as-fabricated pc-LED devices based on YCAS:Cr^{3+} phosphor powders with different Cr^{3+} concentrations as a function of driving current. Note that the NIR output starts to saturate at 300 and 250 mA for the devices based on YCAS:0.04Cr^{3+} and YCAS:0.08Cr^{3+}, respectively.

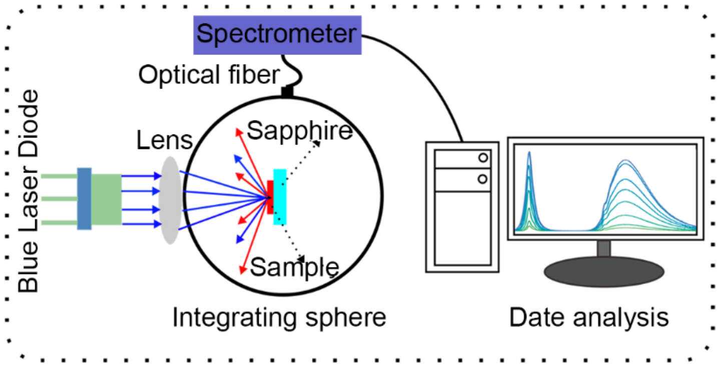

Figure S16 The schematic of the measurement system of laser-driven NIR light sources in a reflective mode.

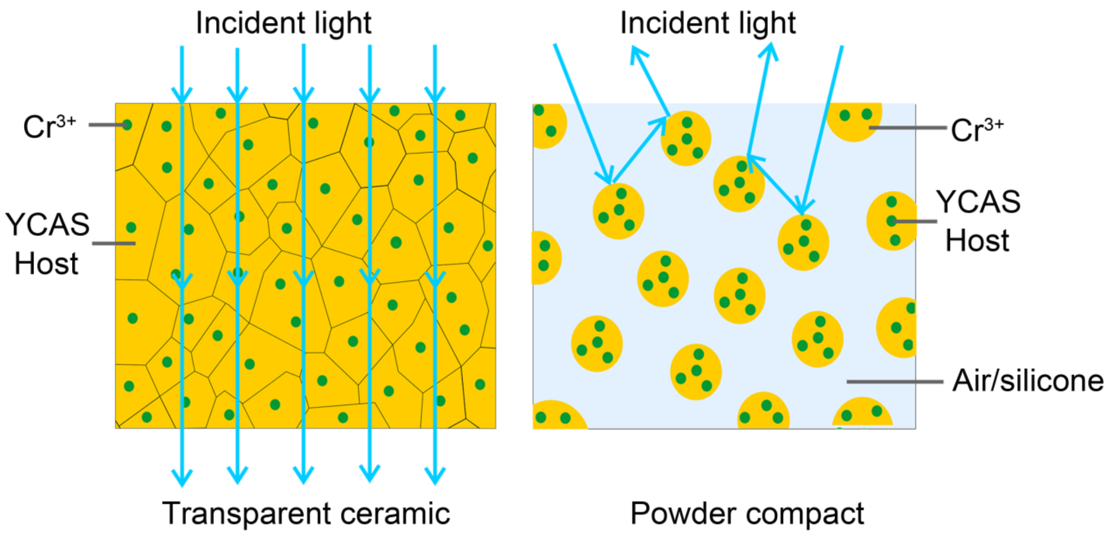

Figure S17 Illustration of the light scattering in transparent ceramic (left) or powder compact (right). For a highly transparent ceramic, there is no scattering center and the incident light can travel longer length within the ceramic, leading to higher probability of encountering the luminescence centers (Cr^{3+}) and thus being absorbed. However, for highly scattering powder compact, the incident will be strongly scattered back.

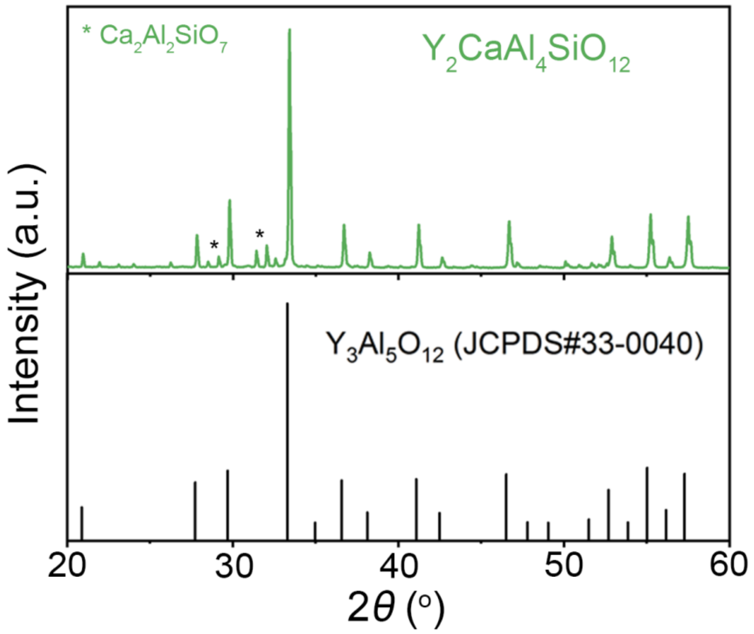

Figure S18 The XRD pattern of $Y_3CaAl_5SiO_{12}$:0.04Cr^{3+} phosphor powders prepared at 1400 ^oC. The secondary phase is mainly $Ca_2Al_2SiO_7$.

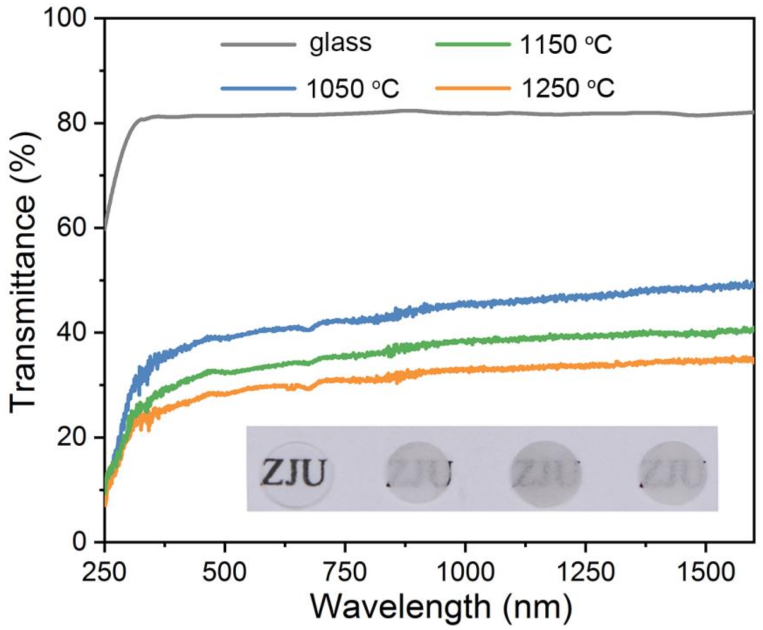

Figure S19. *The transmission spectra of YCAS glass and ceramics (0.4 mm in thickness) annealed at different temperatures.*

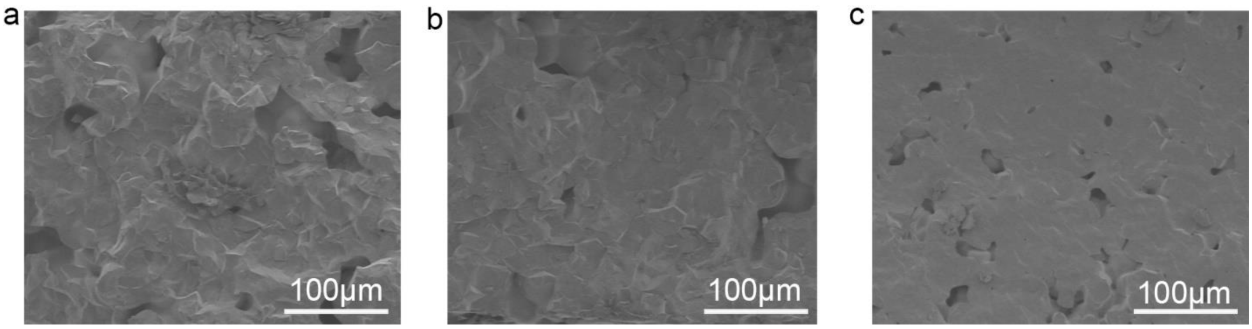

Figure S20. SEM images of the fresh fracture surface of YCAS:Cr^{3+} ceramics annealed at 1050 ^{o}C (**a**), 1150 ^{o}C (**b**) and 1250 ^{o}C (**c**).

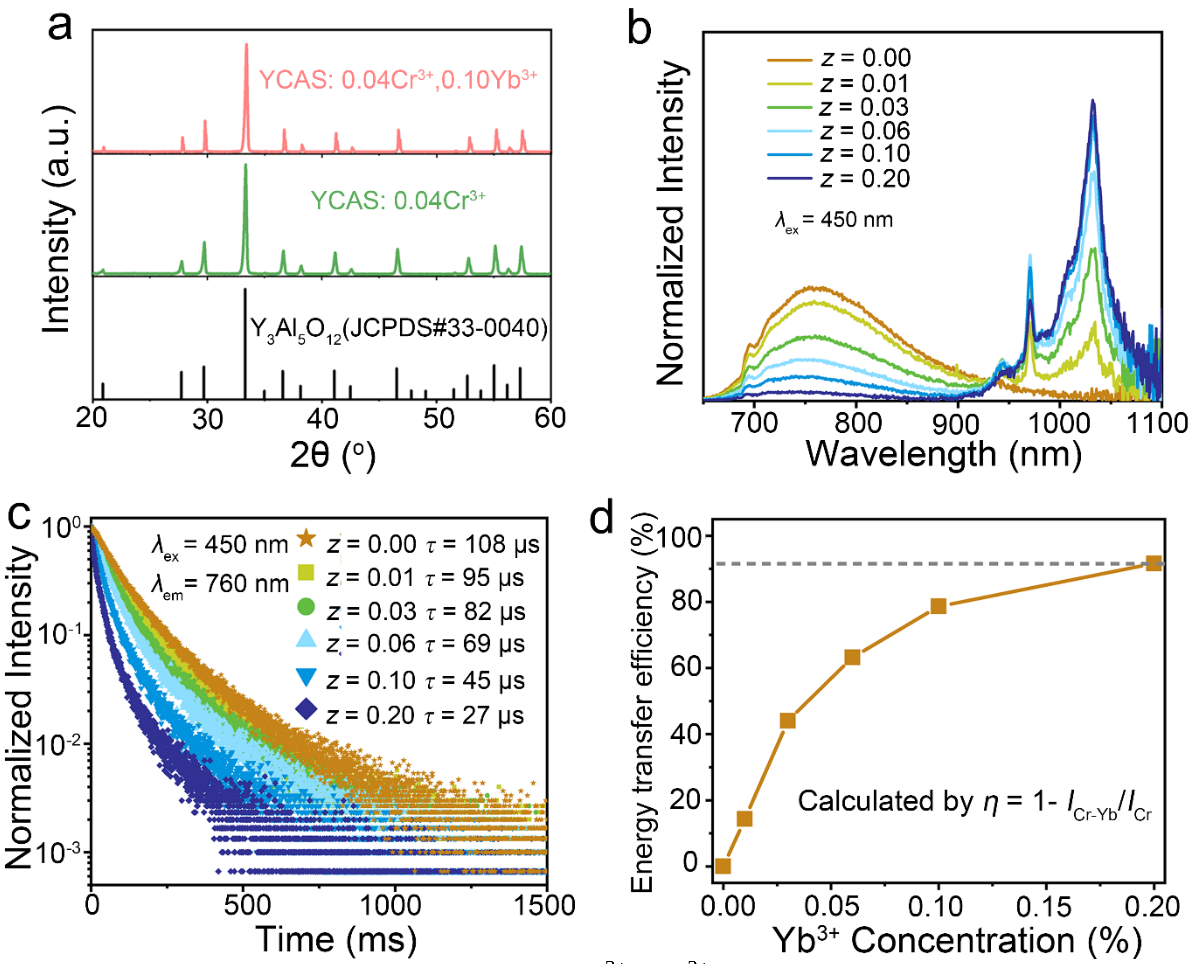

Figure S21. (**a**) XRD patterns of YCAS:0.04Cr^{3+},$z$$Yb^{3+}$ ceramics. (**b**) The emission spectra of YCAS:0.04Cr^{3+},$z$$Yb^{3+}$ ceramics (z = 0.00–0.20) annealed at 1250 °C. (**c**) The decay curves of Cr^{3+} emission (760 nm) YCAS:0.04Cr^{3+}, $z$$Yb^{3+}$ ceramics after pulse excitation at 450 nm. (**d**) The dependence of ET efficiency (η) on Yb^{3+} concentration.

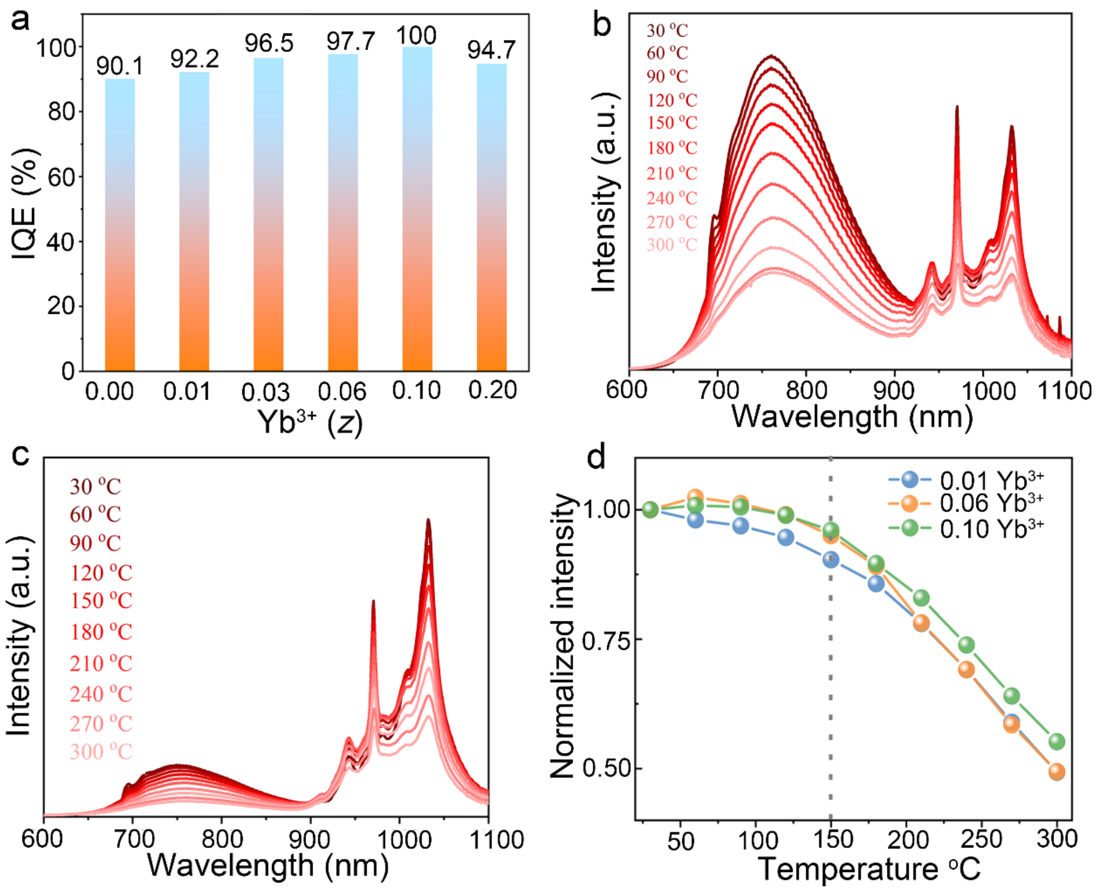

Figure S22. (**a**) The IQE of YCAS:0.04Cr^{3+},zYb^{3+} ceramics annealed at 1250 °C. All of them are higher than 90%. Temperature-dependent spectra of YCAS:0.04Cr^{3+},0.01Yb^{3+} (**b**) and YCAS:0.04Cr^{3+},0.06Yb^{3+} ceramics (**c**) under 450 nm excitation. (**d**) Integrated emission intensities of YCAS:0.04Cr^{3+},zYb^{3+} ceramics (*z* = 0.01, 0.06, 0.10) as a function of the temperature upon 450 nm excitation.

Supplementary Tables

Table S1 *Crystallographic and Rietveld refinement data of* $Y_{3-x}Ca_xAl_{5-x}Si_xO_{12}$ *(x = 0.2, 0.6, and 1.0.)*

Formula	$x = 0.2$	$x = 0.6$	$x = 1.0$
Space group		$Ia\bar{3}d$	
$a = b = c$ (Å)	12.01528(11)	12.00338(10)	11.99011(12)
$\alpha = \beta = \gamma$ (°)	90	90	90
Cell volume (Å^3)	1734.611(29)	1729.461(26)	1723.731(31)
R_p	5.87	5.79	5.58
R_{wp}	8.83	8.67	7.75
Chi2	1.47	1.36	1.12

Table S2 *Wyckoff positions, atomic coordinates and occupancies of $Y_{3-x}Ca_xAl_{5-x}Si_xO_{12}$ (x = 0.2, 0.6, and 1.0.)*

x value	0.2					
Atom	Y	Ca	Si	Al1	Al2	O
Wyck.	24c	24c	24d	16a	24d	96h
Occupancy	0.9333 (0.233)	0.0667 (0.017)	0.0667 (0.017)	1	0.9333 (0.233)	1
Beq	1.427 (19)	1.427 (19)	1.760 (17)	1.465 (45)	1.760 (36)	1.573 (50)
X	0.125	0.125	0.375	0	0.375	0.96833(13)
Y	0	0	0	0	0	0.04923(17)
Z	0.25	0.25	0.25	0	0.25	0.14889(14)

x value	0.6					
Atom	Y	Ca	Si	Al1	Al2	O
Wyck.	24c	24c	24d	16a	24d	96h
Occupancy	0.8 (0.2)	0.2 (0.05)	0.2 (0.05)	1	0.8 (0.2)	1
Beq	1.388 (14)	1.388 (14)	1.469 (33)	1.46 (43)	1.748 (33)	1.542 (48)
X	0.125	0.125	0.375	0	0.375	0.96708(13)
Y	0	0	0	0	0	0.04964(16)
Z	0.25	0.25	0.25	0	0.25	0.14929(14)

x value	1.0					
Atom	Y	Ca	Si	Al1	Al2	O
Wyck.	24c	24c	24d	16a	24d	96h
Occupancy	0.6667 (0.167)	0.3333 (0.083)	0.3333 (0.083)	1	0.6667 (0.167)	1
Beq	0.932 (10)	0.932 (10)	1.229 (26)	0.813 (28)	1.229 (26)	1.196 (35)
X	0.125	0.125	0.375	0	0.375	0.96645(9)
Y	0	0	0	0	0	0.04901(12)
Z	0.25	0.25	0.25	0	0.25	0.14923(11)

Table S3. The average bond lengths of $Y_{3-x}Ca_xAl_{5-x}Si_xO_{12}$ (x = 0.2, 0.6, and 1.0).

Bond lengths	x = 0.2	x = 0.6	x = 1.0
Y/Ca-O in dodecahedra	2.386	2.390	2.394
Al/Si-O in tetrahedra	1.758	1.743	1.734
Al-O in octahedra	1.924	1.926	1.929

Table S4. EDS analysis of YCAS:$0.08Cr^{3+}$ ceramic annealed at 1250 ^{o}C for 10h.

Element	Nominal value (%)	Atomic Fraction (%) [illegible]	Error (%)
Y	10	13.41	5.62
Ca	5	5.81	1.13
Al	19.6	21.82	4.03
Cr	0.4	0.46	0.12
Si	5	5.72	1.07
O	60	52.78	1.91

Table S5. The calculated values of *Dq*/*B* of $Y_{3-x}Ca_xAl_{5-x}Si_xO_{12}$:$Cr^{3+}$ (x = 0.2, 0.6, and 1.0).

x	Dq (cm^{-1})	B (cm^{-1})	Dq/B
x = 0.2	1664	645	2.58
x = 0.6	1658	669	2.48
x = 1.0	1647	686	2.40

Table S6. Performance comparison of translucent YCAS:0.04Cr^{3+} ceramic developed here with the reported broadband NIR phosphors. λ_{max} represents the peak emission wavelength, AE represents the absorption efficiency, and $I_{150°C}$ is the retained emission intensity when the temperature is increased from room temperature to 150 °C

Composition	λ_{max} (nm)	IQE (%)	AE (%)	$I_{150°C}$ (%)	Ref.
YCAS:Cr^{3+}	760	90.1	66.0	91	This work
$La_3Sc_2Ga_3O_{12}$:Cr^{3+}	818	35	–	~60	S1
$NaScGe_2O_6$:Cr^{3+}	895	40.2	–	~20	S2
$CaLuScGa_2Ge_2O_{12}$:Cr^{3+}	800	–	–	59	S3
K_3ScF_6:Cr^{3+}	770	71.7	–	87.3	S4
La_2MgZrO_6:Cr^{3+}	825	~58	32	53	S5
$Ca_2LuHf_2Al_3O_{12}$:Cr^{3+}	785	–	–	~65	S6
$K_3LuSi_2O_7$:Eu^{2+}	740	15	–	59	S7
$MgAl_2O_4$:Mn^{2+}	825	53	–	40	S8
$LiInSi_2O_6$:Cr^{3+}	840	75	–	77	S9
$LiScP_2O_7$:Cr^{3+}	880	38	55	~20	S10
$Ca_2LuZr_2Al_3O_{12}$:Cr^{3+}	750	69	46	~78	S11
K_2NaScF_6:Cr^{3+}	765	74	–	89.6	S12
$Sr_9Ga_{0.2}(PO_4)_7$:Cr^{3+}	850	66	45	~10	S13
Na_3ScF_6:Cr^{3+}	774	91.5	45	~30	S14
$Ga_{1.6}In_{0.4}O_3$:Cr^{3+}	800	88	50	60	S15
Cs_2ZnCl_4:Sb^{3+}	745	69.9	–	–	S16

Table S7. The values of IQE, AE, and EQE of YCAS:Cr^{3+} phosphor powders (450 nm excitation).

Composition	IQE (%)	AE (%)	EQE (%)
YCAS:0.016Cr^{3+}	80.1	20.5	16.4
YCAS:0.04Cr^{3+}	71.4	29.8	21.3
YCAS:0.08Cr^{3+}	50.7	40.1	20.3

Table S8. Comparison of optical performance of the as-fabricated pc-LED with the reported ones.

Phosphor	λ_{max} (nm)	NIR photoelectric efficiency	Ref.
$Y_2CaAl_4SiO_{12}:Cr^{3+}$	760	21.2%@100 mA	this work
$NaScGe_2O_6:Cr^{3+}$	895	1.86%@100mA	S2
$Ca_2LuHf_2Al_3O_{12}:Cr^{3+}$	785	15.75% @100mA	S6
$K_3LuSi_2O_7:Eu^{2+}$	740	7.1% @100mA	S7
$MgAl_2O_4:Mn^{2+}$	825	2.42%@100mA	S8
$LiInSi_2O_6:Cr^{3+}$	840	17.2%@100mA	S9
$K_2NaScF_6:Cr^{3+}$	765	14.02%@100mA	S12
$Sr_9Ga_{0.2}(PO_4)_7:Cr^{3+}$	850	6%@100 mA	S13

Supplementary References

S1. Malysa, B., Meijerink, A. and Jüstel, T. (2018) Temperature dependent Cr^{3+} photoluminescence in garnets of the type $X_3Sc_2Ga_3O_{12}$ (X = Lu, Y, Gd, La). J. Lumin. *202*, 523-531.

S2. Zhou, X. et al. (2020) An ultraviolet-visible and near-infrared-responded broadband NIR phosphor and its NIR spectroscopy application. Adv. Opt. Mater. *8*, 1902003.

S3. Bai, B., Dang, P., Huang, D., Lian, H. and Lin, J. (2020) Broadband near-infrared emitting $Ca_2LuScGa_2Ge_2O_{12}$:Cr^{3+} phosphors: luminescence properties and application in light-emitting diodes. Inorg. Chem. *59*, 13481-13488.

S4. Yu, H., Chen, J., Mi, R., Yang, J. and Liu, Y. (2021) Broadband near-infrared emission of K_3ScF_6:Cr^{3+} phosphors for night vision imaging system sources. Chem. Eng. J. *417*, 129271.

S5. Zeng, H., Zhou, T., Wang, L. and Xie, R. J. (2019) Two-site occupation for exploring ultra-broadband near-infrared phosphor—double-perovskite La_2MgZrO_6:Cr^{3+}. Chem. Mater. *31*, 5245-5253.

S6. He, S. et al. (2020) Efficient super broadband NIR $Ca_2LuZr_2Al_3O_{12}$:Cr^{3+},Yb^{3+} garnet phosphor for pc-LED light source toward NIR spectroscopy applications. Adv. Opt. Mater. *8*, 1901684.

S7. Qiao, J., Zhou, G., Zhou, Y., Zhang, Q. and Xia, Z. (2019) Divalent europium-doped near-infrared-emitting phosphor for light-emitting diodes. Nat. Commun. *10*, 5267.

S8. Song, E. et al. (2019) Heavy Mn^{2+} doped $MgAl_2O_4$ phosphor for high-efficient near-infrared light-emitting diode and the night-vision application. Adv. Opt. Mater. *7*, 1901105.

S9. Xu, X., Shao, Q., Yao, L., Dong, Y. and Jiang, J. (2020) Highly efficient and thermally stable Cr^{3+}-activated silicate phosphors for broadband near-infrared LED applications. Chem. Eng. J. *383*, 123108.

S10. Yao, L. et al. (2020) Enhancing near-infrared photoluminescence intensity and spectral properties in Yb^{3+} codoped $LiScP_2O_7$:Cr^{3+}. Chem. Mater. *32*, 2430-2439.

S11. Zhang, L. et al. (2018) A high efficiency broad-band near-infrared $Ca_2LuZr_2Al_3O_{12}$:Cr^{3+} garnet phosphor for blue LED chips. J. Mater. Chem. C *6*, 4967-4976.

S12. Song, E. et al. (2021) Cr^{3+}-doped Sc-based fluoride enabling highly efficient near infrared luminescence: a case study of K_2NaScF_6:Cr^{3+}. Laser Photonics Rev. *15*, 2000410.

S13. Zhao, F., Cai, H., Song, Z. and Liu, Q. (2021) Structural confinement for Cr^{3+}

activators toward efficient near-infrared phosphors with suppressed concentration quenching. Chem. Mater. *33*, 1c00441.

S14. He, F. et al. (2021) A general ammonium salt assisted synthesis strategy for Cr^{3+}-doped hexafluorides with highly efficient near infrared emissions. Adv. Funct. Mater. *31*, 2103743.

S15. Zhong, J. et al. (2021) Efficient and tunable luminescence in $Ga_{2-x}In_xO_3:Cr^{3+}$ for near-infrared imaging. ACS Appl. Mater. Inter. *13*, 31835-31842.

S16. Su, B., Li, M., Song, E. and Xia, Z. (2021) Sb^{3+}-doping in cesium zinc halides single crystals enabling high-efficiency near-infrared emission. Adv. Funct. Mater. *31*, 2105316.
